# On the Treatment of Croup

**Published:** 1831

**Authors:** 


### ON THE TREATMENT OF CROUP. By MR. GOODLAD.

A PAPER under the above title has recently been published in our new contemporary, the North of England Medical Journal, by a very judicious and able practitioner, Mr. Goodlad, of Bury, which we shall here give some account of, in order that the information contained in it may have as extensive a circulation as our Journal can give it.

Mr. G. deems it unnecessary, of course, to detail the symptoms of croup, a disease which appears to be very prevalent in the district where he resides.

He confines himself, therefore, to a brief outline of the treatment which, in his practice, has almost invariably been found efficient.

"In this complaint," says he, "every gradation may be traced (by placing the ear within a short distance of the patient's chest) from the brazen-like sonorous cough, to the gentle stridule heard more faintly as the patient approaches to convalescence, *and is able to draw a full and a slow inspiration*. On the other hand, if there be rattling conjoined with a disposition to sleep, pale or livid lips, and cold extremities, effusion has taken place—death must follow, and will be accelerated by the remedial means we should otherwise employ. But so long as the attack is accompanied with a high sounding cough, without rattling, the mischief, whatever it may be, is not irremediable; I would go much further, and declare it ought to be arrested, and feel a perfect conviction, in the majority of cases, that it may."

We think that Mr. Goodlad would be more accurate in his diagnosis of the actual state of things, if he put his ear in direct contact with the thoracic parietes; for assuredly he would thus become better acquainted with the natural morbid sounds in the aerial tubes.

Mr. G. observes that there are instances on record where death took place in croup, and where no effusion could be observed—facts which militated in favour of spasm, and probably prevented active treatment. Such instances are, no doubt, rare.

"Amongst the poorer classes in Lancashire, who seldom call in assistance for Croup, until all chance of recovery is destroyed, I have never yet seen a case terminate fatally, without an effusion in the bronchia being conspicuous several hours before death; and I am much disposed to attribute such an event, where it has happened, to the remedies made use of, rather than consider it a natural termination of the disease; particularly as some of those commonly in use are well calculated to produce it.

Amongst these the warm bath is one of the most active, and, at the same time, most injurious; and I cannot imagine how any one, who has once witnessed its effects, can again recommend it in Croup. It is, in my opinion, so decidedly hurtful, by quickening the circulation, that I should interdict its use in almost all inflammatory cases. The warm-bath, I think, is never useful unless prolonged until faintness is produced; and in the early stages of inflammatory complaints, it is often impossible to produce this effect, until the heart beats more than 130 times in a minute, which is a degree of excitement I think unwarrantable. If resorted to later, effusion is brought on sooner than it would otherwise supervene; and many practitioners could, I think, call to mind cases, where its use has been followed by unexpected death: the vessels previously emptied perhaps by bleeding, having given way, and apoplexy supervened."

We have had numerous opportunities of witnessing the injurious effects of inconsiderate recourse to the warm-bath in inflammatory affections; and we, therefore, recommend to the attention of practitioners the foregoing observations of Mr. Goodlad.

Emetics are condemned by our author, and not without reason, as a general remedy in croup. Nauseating doses of emetic tartar are considered as doubtful remedies in the same disease. Blisters in the neighbourhood of the trachea were found to be injurious in the early stages of croup. A heated and close atmosphere in the room of the patient is decidedly injurious.

"From what has already been said, it is evident that two indications are necessary to be attended to, in the cure of Croup; the first is to subdue the inflammation of the windpipe, the other to relieve the oppressed circulation. Without the first object be attained no means will avail; nor will it in every case be safe to wait until that can be accomplished before we relieve the system at large. Danger may be imminent from either of these causes, and we have often to determine whence it is most so, and to regulate our practice accordingly.

The causes which produce Croup, its symptoms and progress, alike indicate the necessity of bloodletting, and this remedy, in comparison with which all others sink into insignificance, should be immediately resorted to. Any quantity of blood may be drawn by leeches, and the local complaint, in almost all cases, be subdued by them; for if one crop of leeches do not remove it others must follow, until the breathing becomes free, or the child so faint, that further depletion would be unsafe. This mode of taking blood, by emptying the vessels which are inflamed, will, it is evident, afford relief, with least expense to the constitution: but when the complaint has existed many hours, and the jugular vein becomes alternately distended and collapsed, during each inspiration; when the angles of the mouth are drawn downwards, every muscle of the neck brought into action, and the breathing consists of a series of gaspings, there will not be time afforded for leeches, and not a moment must be lost. The external jugular vein should be immediately opened with the lancet, though this operation is sometimes exceedingly difficult, requiring a quick eye and a prompt hand to catch it between each inspiration. The struggles of the patient, and the great contraction of the muscles, add to this difficulty; but no consideration should deter us from giving instant relief, and no other method of taking blood seems to afford the same immediate benefit both to the head and breathing. The child may be on the brink of effusion, and every minute lost is matter of serious reproach; but this urgency of the case, which, if not attended to, will speedily be followed by stupor, and that loss of sensibility over the whole frame so favourable to effusion, renders additional precaution necessary; for if the depletion be carried too far, or the vessels emptied very suddenly, that event, so much to be dreaded, will be accelerated.

The finger should therefore be kept upon the pulse whilst the blood is flowing, and the further flow of blood prevented, if the breathing be properly relieved, before faintness is induced. It is safer to trust the further treatment of the case to leeches, which are indeed often necessary even when the jugular vein has been opened, and the loss of blood carried for the time to the greatest extent. This will not be matter of surprise, when we consider how little connexion there is between the arteries ramified upon the inner surface of the windpipe, and the external jugular vein. It is safest, therefore, to unload the general circulation, where that is requisite, from the system at large; and treat the local complaints with leeches where they can be easily obtained; but if not, the finger may be placed upon the orifice for a short time, when the breathing is relieved: and another and a smaller quantity of blood be taken from the same orifice, until faintness deter us from proceeding further."

Mr. G. generally directs leeches to the lower part of the trachea, below the larynx, because they bleed quite as well as on the upper part of the tube, and the blood is thus drawn from those vessels that have most recently taken on morbid action. In whatever way blood be taken, faintness must be induced, and *kept up for some time*.

"It is now that the ear of the practitioner will be most useful to him, and the sound of the cough, the noise which is made by the air passing through the inflamed part, and the frequency and freedom of the inspirations must be closely attended to. He should never leave the bedside of the patient until he is satisfied on every one of these points, since he cannot do so with safety, or consistently with that duty we all owe, where the life of a fellow-creature is at issue. By and bye, he will be rewarded by hearing the cough alter its tone, it becomes loosened, there is a little expectoration, and the child is safe."

A stridule, Mr. G. observes, will remain after the respiration has become free; and neither this symptom nor the high-sounding cough afford sufficient reason for more leeching, yet the long continuance of either of them is an object of suspicion, and *unless the inspiration be free, full, and slow*, we may be assured that inflammation is not entirely removed.

Croup is no doubt an idiopathic disease in many cases, but in the majority, and in the most severe instances, it is accompanied, if not produced, by dentition, together with determination to the head, and a disposition to effusion there. The state of the gums ought, therefore, to be examined in all cases; and they should be freely lanced if they shew heat or thickness in any part. The bowels are to be opened by castor oil or jalap and calomel. The only other medicine which Mr. G. is in the habit of giving is calomel and opium, in large and frequent doses. This combination, before the loss of blood, would be injurious; but after that measure, when the head is free, and the breathing quiet, it produces the best effects. It induces sleep, appeases the cough, determines to the surface, and prevents re-action; whilst the calomel acts here, as in iritis, by preventing effusion and producing absorption.

"Another advantage arising from the combination of opium is, that it enables us to give a larger quantity of calomel, than would be otherwise practicable without its passing off by the bowels; and as the glandular system in children is seldom affected by it, and ptyalism, therefore, rarely induced, we need not be deterred from giving it largely, and have occasion only to watch its operation on the bowels."

Several cases are detailed by Mr. Goodlad in illustration; but the precepts of treatment are so clearly laid down, that we deem it unnecessary to quote any of them in this place. Mr. Goodlad is evidently a decided and judicious practitioner.

---

